# Cross-sectional study on bovine mastitis and its associated risk factors in Ambo district of West Shewa zone, Oromia, Ethiopia

**DOI:** 10.14202/vetworld.2017.398-402

**Published:** 2017-04-17

**Authors:** Edilu J. Sarba, Getachew K. Tola

**Affiliations:** Department of Veterinary Laboratory Technology, College of Agriculture and Veterinary Sciences, Ambo University, PO Box. 19, Ambo, Ethiopia

**Keywords:** Ambo district, California mastitis test, dairy cows, mastitis, prevalence, risk factors

## Abstract

**Aim:**

A cross-sectional study was conducted to estimate the prevalence and associated risk factors of mastitis in dairy cows.

**Materials and Methods:**

A total of 302 dairy cows were selected from all volunteer dairy farms in Ambo district of West Shewa Zone, Oromia region. Thorough clinical examination was made on all lactating cows for evidence of signs of clinical mastitis followed by collection of milk sample for examination of gross changes of milk secretion and California mastitis test.

**Result:**

About 126 (41.7%) cows had mastitis, of which 9.9% (30/302) were clinical and 32.8% (96/302) were subclinical mastitis cases. The quarter level prevalence was 44.4% (536/1208), comprising 9.3% (112/1208), clinical and 32.8% (396/1208) subclinical forms of mastitis. In addition, 5.5% (66/1208) of teats were found to be blind on the clinical examination of udder and teat. The Chi-square analysis of intrinsic risk factors revealed significantly (p<0.05) higher prevalence of mastitis in crossbred cattle (47.2%) than indigenous (15.4%), in cattle above 7 years (75%) than less than 2-6 years of age (28%) and cows given more than 4 calves (81.3%) than those with less than 4 calves (31.1%) irrespective to their lactation stage. There was also significantly (p<0.05) higher mastitis prevalence in larger (46.6%) than smaller herds (24.2%) and among the farming systems in semi-intensive (47.1%) and intensive (42.3%) than extensive (8.1%) management system.

**Conclusion:**

This study indicated a higher prevalence of mastitis linked with several risk factors. Thus, early diagnosis and regular screening of cows for subclinical mastitis together with proper therapeutic management of clinical cases are of paramount importance. Moreover, control and prevention strategies should be designed and implemented with great emphasis given to risk factors to reduce bovine mastitis and its impact on milk production and food security.

## Introduction

Mastitis (inflammation of mammary gland) causes a great deal of loss or reduction of milk yield to influence the development of the dairy industry. It is a complex disease of multifactorial etiology caused by a variety of microorganisms including bacteria, fungi, and algae. The majority of mammary infections are caused by bacteria namely staphylococci, streptococci, and enterobacteriacae. Organisms such as coagulase-negative staphylococci, environmental streptococci, *Mycoplasma* species, and *Serratia* spp. are increasingly implicated as emerging pathogens causing mastitis [1]. Mastitis can be classified as clinical and subclinical. Clinical mastitis is characterized by change in the morphology of the udder, chemical and physical changes in the milk, while the subclinical form is without any noticeable manifestations of inflammation. Subclinical mastitis is more common than the clinical mastitis and causes the greatest overall losses in most dairy herds [2].

Several scholars agree that mastitis is one of the most costly diseases of dairy industry worldwide. It is estimated that on average an affected quarter suffers 30% reduction in productivity and an affected cow loses 15% of its production for the lactation [1,3]. The economic implication of bovine mastitis is derived from the high costs of treatment and diagnosis, loss of milk production, early culling, and cost of the control program in clinical and subclinical cases. A large proportion of mastitis is not detectable only by clinical examination of the udder and milk that poses economic loss from decreased production, which makes it difficult to estimate the economic losses [4]. In addition, the bacterial contamination of milk from affected cows may render it unsuitable for human consumption due to zoonosis, food poisoning, and antibiotic residue in the milk following mastitis [1].

In Ethiopia, even if it is not well established, several studies in various parts of the country were conducted on bovine mastitis recently [5-8]. However, more research outputs are needed on the epidemiology of the disease in many areas of the country including Ambo to support the control and prevention strategies of this economically important disease. Bovine mastitis is one of the diseases the country’s dairy farm personnel, particularly Ambo district livestock experts and dairy owners complain about its being the cause of culling and reduction of milk production. At present, there is a growth of modern dairy farming in urban and peri-urban areas of major towns in Ethiopia. Moreover, the milk production in Ethiopia is expected to increase more rapidly in response to the fast growing demand for milk and milk products resulting from increasing human population in urban areas, and rising consumer income [9]. Thus, it is necessary to have epidemiological information about mastitis and factors associated with udder infection so as to improve dairy production and uphold quality of milk for consumers.

Therefore, the objectives of this study were to estimate the prevalence of the clinical and subclinical mastitis and to identify the potential risk factors in dairy cows in Ambo district.

## Materials and Methods

### Ethical approval

All animals in this study were treated according to the ethical standards of Ambo University, and all animal handling was assessed and approved by the Ambo University Animal Research Ethics Review Committee. The farmers were informed about the purpose and the methods of the study. Oral consent was obtained from each farmer before commencement of the study.

### Study area

The study was conducted in Ambo district of West Shewa zone, Oromia Regional State, from November 2013 to July 2014. Ambo town is the administrative center of the Zone located 114 km west of Addis Ababa at latitude of 8°59′N 37°51′E and longitude of 8.983°N 37.85°E. The elevation of Ambo district ranges from 1900 to 2275 meters above sea level. Its temperature ranges from 19°C to 29°C with average of annual temperature of 22°C and the average annual rainfall of about 900 mm. The number of cattle in the area is estimated to be around 133, 202 [10].

### Study animals

Study animals were dairy cows including cross breed and indigenous zebu cows. The *Horro* is the dominant indigenous breeds in the area followed by short horn highland breed. Both are managed under extensive system. Most of the crosses in the area are the hybrids of these indigenous breed and Holstein-Friesian and are reared either under an extensive, intensive, or semi-intensive system.

### Study design and sample size determination

A cross-sectional study was used to screen mastitis in lactating dairy cows. The sample size was determined according to Thrusfield [11] formula with expected prevalence of 74.4% [12] and 95% statistical confidence level. Accordingly, 290 lactating cows were intended to be examined; however for the purpose of this study, a total of 302 lactating cows were included in the study. In general, 25 dairy farms of which 17 were small-holders (owning <10 cows) and 9 were from medium-sized (owning >10 cows) owned by volunteer owners were included in the study. The udder and teats of all lactating cows in the selected farm were clinically examined and milk samples were collected for California mastitis test (CMT).

### Data collection

Data collection format was prepared and used to record age, breed, parity, and lactation stage of cows at the same time while milk samples were taken. Age of the animals was determined based on birth records and dentition and grouped as young adults (2-6 years) and adults/old (≥7 years). Stage of lactation was categorized as early (<5^th^ month) and late (>5^th^ month). Parity was also categorized as few (with 1-4 calves) and many (≥5 calves). Information on extrinsic factors such as management system (as extensive, intensive, and semi-intensive), herd size (as <10 cows and >10 cows), and education status (as illiterate and literate) was also gathered.

### Clinical examination of udder

The udders were carefully inspected followed by thorough palpation to detect possible fibrosis, inflammatory swellings, visible injury, tick infestation, atrophy of the tissue, and swelling of supramammary lymph nodes. The size and consistency of mammary quarters were checked for the presence of abnormalities such as disproportional symmetry, swelling, firmness, and blindness. Viscosity and appearance of milk secretion from each mammary quarter were examined for the presence of clots, flakes, blood, and watery secretions [13].

### CMT

The collected milk samples were screened by the CMT according to Quinn *et al*. [14]. From each quarter of the udder, a squirt of milk was placed in each of the cups on the CMT paddle, and an equal amount of 3% CMT reagent was added to each cup and mixed well. The result of CMT was based on the nature of coagulation and viscosity of the mixture which show the presence and severity of the infection, respectively [6]. Mastitis detection was made based on CMT result for subclinical cases. Accordingly, the degree of coagulation was graded as “–” (negative), “+” (Slightly positive), “++” (Moderately positive), and “+++” (Highly positive). Results graded above slightly positive were all considered as CMT positive. In addition to the CMT result, milk with pus flakes, clots, or blood-tinged watery secretion, yet no visible or palpable changes in mammary quarters, and acute mastitis with signs of inflammation and or systemic involvement were diagnosed as clinical mastitis. Mastitis was categorized as clinical if lactating cows exhibited clinical features of mastitis, or subclinical based on degree of coagulation on examination using CMT.

### Data management and analysis

Data entry and management was made using Microsoft Excel 2007, while data analysis was performed using SPSS-17 Software. Descriptive statistics was performed to summarize the prevalence of mastitis and blind teat. The Chi-square test was used to assess the association of potential risk factors with the prevalence of mastitis. In all the analyses, confidence level of 95% and significance level of α≤0.05 were used.

## Results

### Over all prevalence of mastitis

Of the total 302 examined lactating cows, 126 cows (41.7%) cows were found to be positive for mastitis. Out of this, 30 (9.9%) were clinical mastitis and 96 (31.8%) subclinical mastitis cases. Of 1208 quarters examined, 66 (5.5%) teats were found blind. From the functional 1142 teats examined, 112 quarters (9.3%) showed clinical mastitis. From those teats screened by CMT, 384 quarters (31.8%) showed evidence of subclinical mastitis (Table-1).

**Table-1:** The prevalence of mastitis at individual animal and quarter level in dairy cows of Ambo district.

Disease status	Number of animals examined	Number of positive (%)	95% CI	Number quarter examined	Number of positive (%)	95% CI
Clinical mastitis	302	30 (9.9)	5.1-14.8	1208	112 (9.3)	7.0-11.6
Subclinical mastitis	302	96 (31.8)	24.3-39.3	1208	384 (31.8)	28.6-35.5
Blind teats	-	-	-	1208	66 (5.5)	3.7-7.3
Total	302	126 (41.7)	33.8-49.7	1208	562 (46.5)	

CI=Confidence interval

### Risk factors

As shown in Table-2, this study revealed significantly higher prevalence of mastitis (47.2%; n=112) in cross breeds as compared to local breeds (15.4%; n=8) in (p<0.05). With respect to age, there was an observation of significantly higher (p<0.005) prevalence in old cows (70.5%; 62/88) than in young cows (28.0%; 60/214). There was also a significant association between parity and mastitis evidenced by higher infection rate of 81.3% (52/64) in cows having more than 4 calves 31.1% (74/238) than in cows having less than 4 calves (Table-2). In contrary, the prevalence of mastitis was not significantly influenced by stage of lactation, though there is higher prevalence of in early stage (45.9%; 68/148) than in late stage (37.7%; 58/154).

**Table-2:** Prevalence of mastitis in milking cows based on intrinsic and extrinsic factors.

Risk factors	Category	Number of animals examined	Number of positives (%)	X^2^	p value
Breed	Cross	250	112 (47.2)	8.960	0.003*
	Local	52	8 (15.4)		
Age	Young adult (2-6 years)	214	60 (28.0)	28.281	0.000*
	Adult (≥7 years)	88	62 (70.5)		
Parity	Few (<4 calves)	238	74 (31.1)	26.093	0.000*
	Many (>4 calves)	64	52 (81.3)		
Lactation stage	Early (<5 month)	148	68 (45.9)	1.065	0.302
	Late (>5 month)	154	58 (37.7)		
Herd size	Small (<10 cows)	66	16 (24.2)	5.306	0.021*
	Large (>10 cows)	236	116 (46.6)		
Educational status	Illiterate	90	40 (44.4)	0.195	0.658
	Literate	212	86 (40.6)		
Management system	Extensive	24	2 (8.1)	6.307	0.043*
	Semi-intensive	136	64 (47.1)		
	Intensive	142	60 (42.3)		
Tick control activity	Yes	94	38 (40.4)	0.047	0.828
	No	208	88 (42.3)		

χ^2^=Chi-square.

* p<0.05=Significant

Analysis of extrinsic risk factor showed higher infection rate in animals from larger herds (46.6%) than small herd (24.2%). In comparing prevalence among management systems, the highest infection rate was observed in semi-intensively managed cows (47.1%) followed by cows in intensive management system (42.3%) and the least in extensively managed cows (8.1%). Both herd size and management systems were found to be significantly associated with the udder infection (p<0.05). Nevertheless, there was no statistically significant (p>0.05) difference in prevalence between animals owned by illiterate (44.4%) and literate (40.6%) owners; the same is true for owners practicing tick control (40.4%) and those who did not (42.3%).

## Discussion

This study showed the overall prevalence of mastitis in dairy cows in Ambo district to be 41.7%, which is comparable with 44.1% prevalence reports of Delelesse [15] from Ethiopia. However, it is relatively lower than the report of 58% by Lidet *et al*. [5], 52.27% by Fufa *et al*. [6], and 52.6% by Tadesse *et al*. [7]. On the other hand, Endale *et al*. [8] reported 32.92% prevalence which was lower than the present finding. The different reports are from different management systems, breeds of cattle and agro-climatic areas, thus this could contribute to the variability of mastitis prevalence among reports.

The finding of 9.9% prevalence of clinical mastitis and 31.8% prevalence of subclinical mastitis in this study is in accordance with the view of scholars that subclinical mastitis is 3-4 times more frequent than clinical mastitis [1]. The study closely agrees with the reports of Abera *et al*. [16], Mekibib *et al*. [17], and Haftu *et al*. [18]. However, it was lower than the report of Tafa *et al*. [19] and Duguma *et al*. [20]. The higher prevalence of subclinical mastitis might be owed to the strong cows’ udder defense mechanism according to Jha *et al*. [21], whereas the lower prevalence of clinical mastitis is attributed to treatment after the appearance of the clinical signs. Moreover, the higher prevalence of subclinical mastitis in the study area could be attributed to the little attention given to it and farmers are not aware about the silent cases of mastitis.

High prevalence of blind mammary quarters (5.5%) closely agrees with the result of Biffa *et al*. [13]. A lack of screening subclinical mastitis and late or not treating clinical cases could possibly leads to blindness of mammary gland. Blind mammary quarters contribute to high subclinical mastitis and loss of milk production with a subsequent impact on food security. The quarter level prevalence of clinical (9.3%) and subclinical (31.8%) mastitis in this study indicates the economic significance of the disease [22]. The relatively higher prevalence of mastitis in right front quarters (21.3%) and left hind quarters (22.4%) in this study agrees with the findings of other scholars [12,22]. This might be due to ease of grasping the right front quarters first while milking and the higher production capacity of hind quarters [1], and the chance of getting environmental and fecal contamination in the case of hind quarters [22].

According to Radostitis *et al*. [1], the higher susceptibility of higher-yielding cows to mastitis could be attributed to the anatomy of teat and udder and certain physiological characteristics such as fewer phagocytic cells in higher yielding cows associated to dilution. In line with this, it was found in this study that the prevalence of mastitis in crossbred cows (47.2%) was significantly higher (p<0.05) than in local cows (15.4%). Significantly higher prevalence of mastitis (75%) in older than young adult cows (28.0%) was in agreement with Fufa *et al*. [6] who reported increased the prevalence of mastitis with advancing lactation number. Jha *et al*. [22] explained the contribution of better active mononuclear leukocyte function in primiparous cows than the multiparous cows. This is in agreement with our current finding of significantly higher prevalence of mastitis in cows with many calves (81.3%) than with few calves (31.1%). Moreover, cows with advanced parity become more productive, so it can be assumed that as the parity of cow advances and the age increases cows become prone to mastitis.

The rate of infection revealed significant variation (p<0.05) between small herd size (24.2%) and large herd size (46.6%). This indicates that besides the lack of knowledge of the community regarding animal husbandry the large size can affect the health of animals. A wide range of farm and animal level management factors can influence husbandry conditions [13]. This was obvious in this study, which showed significant higher prevalence in semi-intensive (47.1%) and intensive farming systems (42.4%) when compared to extensive farming systems (8.1%). This could be attributed to the variation in hygienic standards of dairy environment and milking conditions as the cows in these systems in this study were maintained in a dirty and wet area which favors the proliferation and transmission of mastitis causing organisms. In conjunction with this, the questionnaire finding indicated that the majority of the dairy farms were not visited by veterinarians and training for farm owners was not practiced at all, which could negatively influence dairy health management in the area.

Under Ethiopian condition all dairy farmers do not exercise management practices such as teat dipping, milking with gloves, machine milking and also the do not allow calves to suckle in cross breed dairy cattle. In addition, milk yield of the cows is not considered in this study, because farmers are reluctant to tell the actual yield of their cows, as it is believed to be bad practice by the local community. However, management practices such as pre-milking udder cleaning and bedding material were unintentionally overlooked in this study.

## Conclusion

The present study indicated mastitis is a prevalent disease in dairy cows in Ambo district. Breed, age, parity, herd size, and management system are the main factors that were associated with mastitis. The subclinical cases are by far more frequent than the clinical ones. Unequivocally, silent loss of milk production due to subclinical mastitis along with blind teats could cause adverse effect on the dairy activity of the town. This implies that mastitis has an overlooked impact on dairy development and food security in the area. Therefore, awareness should be created to ensure early diagnosis and regular screening of cows for subclinical mastitis together with the treatment of clinical cases. Moreover, mastitis control and prevention strategies should be designed and implemented with consideration of the age, parity of cows and management system so as to reduce its influence on milk production and food security. Further detailed epidemiological, microbiological, and economic analysis studies are suggested at countrywide level to shape the existing control and prevention strategies.

## Authors’ Contributions

GKT designed and conducted the research work. Data were analyzed and the manuscript was written by EJS and GKT. Both the authors have read and approved the final manuscript.

## References

[1] Radostits O.M, Gay C.C, Hinchcliff K.W, Constable P.D, Mastitis (2007). Veterinary Medicine:A Text Book of Disease of Cattle, Sheep, Pigs, Goats, and Horses.

[2] Eriskine R.J (2001). Intramuscular administration of ceftiofur sodium versus intra-mammary infusion of penicillin/novobiocin for treatment of *Streptococcus agalactiae* mastitis in dairy cows. J. Am. Vet. Med. Assoc.

[3] Bartlet P, Joust V.W, Devid J.W, Charles D.G (1991). Temporal patterns of lost milk production following clinical mastitis in a large Michigan Holstein herd. J. Dairy Sci.

[4] Brinda M, Moga-Manzat R, Brezovan D, Toth E (2009). Study on correlation between different diagnosis and tests in bovine mastitis. Lucrări Ştiinłifice Med. Vet.

[5] Lidet G.M, Benti D, Feyissa B, Abebe M (2013). Study on prevalence of bovine mastitis in lactating cows and associated risk factors in and around Areka town, Southern of Ethiopia. Afr. J. Microbiol. Res.

[6] Fufa A, Gemechis F, Bekele M, Alemayehu R (2013). Bovine mastitis:Prevalence, risk factors and bacterial isolation in small-holder dairy farms in addis Ababa City, Ethiopia. Glob. Vet.

[7] Tadesse T, Mulisa M.K, Teka F (2014). Bovine mastitis:Prevalence and isolation of major pathogens in dairy farms of selected sites in Addis Ababa, Ethiopia. Appl. J. Hyg.

[8] Endale M, Eyob E, Addisu A, Naod T (2016). A study on the prevalence of bovine mastitis and associated risk factors in and the surrounding areas of sodo Town, Wolaita Zone, Ethiopia. Glob. J. Sci. Front. Res. D Agric. Vet.

[9] GOE LMP (2007). Government of Ethiopia, The Livestock Master Plan Study Report.

[10] CSA, (Central Statistics Agency) (2003). Agricultural Sample Survey 2001/2.

[11] Thrusfield M (2005). Veterinary Epidemiology.

[12] Tesfahewot Z, Aya T, Bayecha R (2013). Study on prevalence, bacterial pathogens and associated risk factors of bovine mastitis in small holder dairy farms in and around Addis Ababa, Ethiopia. J. Anim. Plant Sci.

[13] Biffa D, Debela E, Beyne F (2005). Prevalence and risk factors of mastitis in lactating cows in Southern Ethiopia. Int. J. Appl. Res. Vet. Med.

[14] Quinn P.J, Carter M.E, Markey B, Carter G.R (1999). Clinical Veterinary Microbiology.

[15] Delelesse G.D (2010). Study on prevalence of bovine mastitis on cross breed dairy cow around Holeta areas, West Shoa zone of Oromia, Ethiopia. Glob. Vet.

[16] Abera M, Demsie B, Aragaw K, Regassa F, Regassa A (2013). Isolation and identification of *Staphylococcus aureus* from bovine mastitic milk and their drug resistance pattern in Adama town, Ethiopia. Afr. J. Dairy Farming Milk Prod.

[17] Mekibib B, Furgasa M, Abunna F, Megersa B, Regasa A (2010). Bovine mastitis:Prevalence, risk factors and major pathogens in dairy farms of Holeta town Central Ethiopia. Vet. World.

[18] Haftu R, Taddele H, Gugsa G, Kalayou S (2012). Prevalence and bacterial cause, and antimicrobial susceptibility profile of mastitis isolates from cows in large-scale dairy farms of Northern Ethiopia. Trop. Anim. Health Prod.

[19] Tafa F, Terefe Y, Tamerat N, Zewdu E (2015). Isolation, identification and antimicrobial susceptibility pattern of coagulase positive Staphylococcus from subclinical mastitic dairy cattle in and around Haramaya university, Ethiop. Vet. J.

[20] Duguma A, Tolesa T, Yohannes A (2014). Prevalence of clinical and subclinical mastitis on cross bred dairy cows at Holleta Agricultural Research Center, Central Ethiopia. J. Vet. Med. Anim. Health.

[21] Jha A.K, Hoque M.N, Kamal M.M, Rahman M.M, Bhuiyan M.M.U, Shamsuddin M (2010). Prevalence of mastitis and efficacy of different treatment regimens on clinical mastitis income. SAAR J. Agric.

[22] Sori H, Zerihun A, Abdicho S (2005). Dairy cattle mastitis in and around Sebeta, Ethiopia. Int. J. Appl. Res. Vet. Med.

